# Conserved Phosphorylation of the Myosin1e TH1 Domain Impacts Membrane Association and Function in Yeast and Worms

**DOI:** 10.1002/cm.22026

**Published:** 2025-04-09

**Authors:** Holly R. Brooker, Karen Baker, Marina Ezcurra, Philippe P. Laissue, Lin Wang, Michael A. Geeves, Jennifer M. Tullet, Daniel P. Mulvihill

**Affiliations:** ^1^ School of Biosciences, University of Kent Canterbury Kent UK; ^2^ School of Life Sciences, University of Essex Colchester UK; ^3^ Central Laser Facility, Research Complex at Harwell Science and Technology Facilities Council, Rutherford Appleton Laboratory Oxford UK

**Keywords:** *fission yeast*, *Hum‐1*, *Hum‐5*, *Myo1*, *myosin 1d*, *myosin 1e*, nematode ( *Caenorhabditis elegans*), *plecktrin homology*, *Schizosaccharomyces pombe*, *TOR signalling*

## Abstract

Cells have an intrinsic ability to rapidly respond to environmental change to regulate cell cycle progression and membrane organisation, thereby affecting cell growth and division. The actin cytoskeleton is a highly dynamic complex of proteins that can rapidly reorganise to change the growth pattern of a cell. Class I myosins are monomeric actin‐associated motor proteins that play key roles in diverse cellular functions such as tension sensing and membrane reorganisation, as well as promoting actin polymer nucleation at sites of cell growth. We have analysed the localisation and function of both *C. elegans* class 1 myosins, HUM‐1 (Myo1e) and HUM‐5 (Myo1d). Both motors are non‐essential. While HUM‐1 is expressed in diverse cells and tissues, HUM‐5 localises exclusively to a subset of cells in the nervous system. While animals lacking *hum‐1* displayed a reduced maximal brood size and a delay in embryo release, deleting both *hum‐1* and *hum‐5* together shortened *C. elegans* lifespan. Moreover, we identified that phosphorylation of a conserved serine residue within the Myo1e TH1 domain had an impact on the localisation and function of the motor protein in both *C. elegans* and the fission yeast, *S. pombe*, indicating this modification modulates the ability of Myo1e/HUM‐1 to interact with phospholipids at the plasma membrane. We conclude that TH1 domain phosphorylation plays a key role in regulating the cellular distribution and function of Myo1e motors across all eukaryotes.

## 1 Introduction

Myosins are a superfamily of actin‐associated motor proteins involved in a wide range of cellular processes, including intracellular transport, organelle movement, and cytoskeletal organization. The unconventional class I myosins play specialized roles in cellular processes such as membrane trafficking and actin cytoskeleton remodelling (Girón‐Pérez et al. 2019; McIntosh and Ostap 2016). These single‐headed, actin‐associated ATPase motor proteins contain amino‐terminal motor domains that exert force against actin, a light‐chain‐binding neck region, as well as a tail domain that mediates membrane association and interactions with cellular cargo (McIntosh and Ostap 2016). Unconventional myosin heavy chains are subdivided into sub‐classes on the basis of phylogenetic analysis of the head domain and molecular differences in the neck and tail domains (Mooseker and Cheney 1995). Based on this convention, class 1 myosins are currently subdivided into 6 subclasses, named A to H (Girón‐Pérez et al. 2019). The 1D subclass of myosins (Myo1d) is found in eukaryotic organisms ranging from protozoans to humans. They are associated with sensory structures, such as stereocilia, where they play key roles in cellular mechanosensory and auditory processes (Dürrwang et al. 2006; Adamek et al. 2008; Laakso et al. 2008). They are also associated with a range of human pathologies (Navinés‐Ferrer and Martín 2020). Like Myo1d, the 1E subclass of myosins (Myo1e) is composed of single‐headed, long‐tailed class I myosin motors, but has distinct cellular functions (McIntosh and Ostap 2016). While Myo1d and Myo1e possess subclass‐specific domains within their tail regions, both possess highly conserved lipid‐binding motifs, including a Pleckstrin Homology (PH) domain within the TH1 Tail Homology region (Zhang et al. 2019; Mazerik et al. 2014; Chen et al. 2012), that enables targeting to specific membrane regions where the myosin affects membrane invagination and scission during endocytosis and phagocytosis.

Myosin motors are subject to different types and levels of regulation, each impacting specific aspects of the protein's ability to: (1) associate with, and (2) exert motile force against, actin; (3) interact with light chains; (4) cargoes, and (5) organelles (Heissler and Sellers 2016). These regulatory mechanisms include association with calmodulin‐like light chains; conformation changes of the heavy chain, tropomyosin association with the actin polymer; tail domain‐ligand binding, and phosphorylation of myosin heavy and light chain. The latter is affected by signaling pathways that are activated in response to diverse physiological changes, such as cell cycle timing and extracellular stimuli.

The fission yeast, *Schizosaccharomyces pombe*, and nematode worm, *Caenorhabditis elegans* , are both attractive model systems for studying the dynamic regulation and function of the actomyosin cytoskeleton (Win et al. 2002; Johnson et al. 2022; Kovar et al. 2010). These genetically tractable organisms each allow the study of cytoskeleton organisation and dynamics with different levels of molecular detail. The simple unicellular fission shape lends itself to the detailed study of single molecule dynamics throughout the different lifecycles. *C. elegans* provides a simple animal model for understanding the role specific proteins play in the organisation, development, growth, and viability of a complex multi‐cellular animal. While the yeasts contain a minimal subset of myosin heavy chain classes for unicellular organisms (class I, II and V), the nematode expresses one of the smallest subsets of myosin heavy chains for an animal (classes I, II, V, VI, VII, IX and XII) (Win et al. 2002; Baker and Titus 1997; Johnson et al. 2022; East and Mulvihill 2011). Yeasts express a single class I myosin of E subclass, while *C. elegans* contains two class I myosins, one classified into each of the D and E subclasses (Johnson et al. 2022). Little is currently known about how and where they function within the worms.

Here we present an analysis of both *C. elegans* class I myosin orthologues, HUM‐5/Myo1d and HUM‐1/Myo1e. We show that they have non‐overlapping expression patterns, with each localising to discrete neuronal and reproductive cells within adult worms. While neither protein is essential for viability, HUM‐1/Myo1e is required for controlling brood size and embryo release. Moreover, removal of both myosins significantly reduces the overall lifespan of the animals. Using both fission yeast and nematode model systems, we go on to establish that phosphorylation of a conserved serine within the TH1 domain of the myosin 1e subclass is required for association of the motor protein with the membrane at sites of cell growth. This provides a simple reversible mechanism to regulate the membrane associated function of Myo1e motors across eukaryotes.

## 2 Materials and Methods

### 2.1 Generation of *mNeongreen‐myo1‐S782A* Fission Yeast Strains

DNA containing the *myo1‐S782A and myo1‐*S782*D* phosphomutant alleles was synthesised and cloned using *HpaI‐AvrII* into a previously generated ura4 vector, replacing a portion of the existing wild‐type *myo1* ^+^ gene (Baker et al. 2019). The presence of the *myo1‐S782A* mutation was confirmed by the introduction of a *HindIII r*estriction site. A 500 bp upstream region of the *myo1* ^+^ gene together with mNeongreen cDNA was cloned as an *AflII*‐*AvrII* fragment at the *myo1* ATG start codon (Baker et al. 2019). The DNA was linearised and introduced into *myo1::kanMX6 ura4.d18* cells. Integration at the *myo1* ^+^ locus was identified by marker replacement and subsequently confirmed by PCR and sequencing.

### 2.2 Generation of *C. elegans* Myosin I Reporter Constructs: *gfp::hum‐1*

The *hum‐1* 2 kb upstream promoter region was amplified from *C. elegans* cDNA using PCR and cloned into pGEM‐T‐Easy. A GFP tag (optimized for *C. elegans* use) was synthesized and inserted into a pUC18 plasmid using *BamHI* and *SalI* restriction sites. The promoter region was then excised and inserted into the pUC18‐GFP construct via *SanDI*—*BamHI* digest. Synthesized *hum‐1* was subsequently cloned into the pUC18‐*Phum‐1::GFP* by a *Not I—NruI* digest to create the final construct; pUC18‐*Phum‐1::GFP*::*hum‐1* (referred to as *GFP::hum‐1*). ***gfp::hum‐1‐S734A/D***: The two restriction sites, *Nae1* and *Xho1*, were introduced into the synthesized *hum‐1* gene either side of the region encoding conserved serine 734. The DNA was prepared by *Nae1‐Xho1* digest and ligated with PCR product from oligonucleotides, which encoded the S734A mutation. The introduction of this portion of DNA also introduced an *Mlu1* site for correct clone identification. Once the S734A mutant was attained, it was digested using a *Nae1*—*Xho1* digest and ligated with PCR product from oligonucleotides 566 and 567, which encoded the S734D mutation. Correct clones were identified by loss of *Mlu1* site. ***mCherry::hum‐5***: Two restriction sites, *PvuI* and *NruI*, were introduced into the synthesized *hum‐5* gene. The DNA was prepared by using a *PvuI—NruI* digest and ligated into the synthesized *Phum‐5::mCherry* vector. The DNA was then excised as a *BamHI—SalI* fragment and cloned into a pUC18 based vector. *gfp::hum‐1, gfp::hum‐1S734A, gfp::hum‐1S734D*, or *mCherry::hum‐5* transgenes were introduced into *C. elegans* by co‐injecting the plasmid DNA into the distal arm of the gonad of day one adult wild‐type (N2) nematodes along with the *rol‐6* marker (Mello et al. 1991), and progeny carrying stable, extrachromosomal arrays isolated by presence of rolling animals and fluorescence using low excitation intensity and short exposure times to avoid any risk of potential photodamage.

### 2.3 Generation of Myo1e TH1 Domain Expression Constructs

cDNA encoding the Myo1e TH1 domain were PCR amplified as Nde1—BamH1 fragments from the genome of wild type, *mNeongreen‐myo1‐S782A* and *mNeongreen‐myo1‐S782D S. pombe* cells, as well as wild‐type, *gfp::hum‐1‐S734A* and *gfp::hum‐1‐S734D C. elegans* worms. Each fragment was ligated into the pET151 D‐TOPO plasmid (Invitrogen) together with a 5’ mNeongreen fragment, and sequenced. These TH1 *Nde1—BamH1* fragments were cloned from the subsequent bacterial recombinant expression constructs and ligated into the *S. pombe* episomal thiamine‐repressible construct, pREP41‐NGFP (Craven et al. 1998).

### 2.4 *C. elegans* Husbandry

All lines used in this study were either generated from, or back‐crossed six times with, a laboratory wild‐type stock. *C. elegans* strains used in the study are listed in Table 1. *hum‐1* and *hum‐5* mutant strains were obtained from the Caenorhabditis Genetics Center (CGC). *C. elegans* were maintained using standard culture methods (Brenner 1974). All strains were grown at 20°C on NGM plates with OP50 (Stiernagle 2006). Strains used were kept at 20°C unless otherwise stated. All strains were maintained in a well‐fed and clean state for at least three generations before use in any experiments.

**TABLE 1:** *C. elegans* strains used in this study.

#	Strain	Source
N2	*C. elegans* wild type (ancestral)	Lab stock
RB1557	*hum‐5* (ok1885) III	*CGC*
RB818	*hum‐1* (ok634) I	*CGC*
JMT6	*hum‐1* (ok634) I	This study
JMT8	*hum‐1* (ok634) I; *hum‐5* (ok1885) III	This study
JMT12	*[Phum‐1::gfp::hum‐1 S734D::hum‐1 3′UTR]*	This study
JMT15	*[Phum‐1::gfp::hum‐1 S734A::hum‐1 3′UTR]*	This study
JMT19	*[Phum‐1::gfp::hum::hum‐1 3′UTR]*	This study
JMT24	*hum‐1* (ok634) I; *[Phum‐1::gfp::hum::hum‐1 3′UTR*]	This study
JMT25	*hum‐1* (ok634) I; [*Phum‐1::gfp::hum‐1 S734A::hum‐1 3′UTR*]	This study
JMT26	*hum‐1* (ok634) I; [*Phum‐1::gfp::hum‐1 S734D::hum‐1 3′UTR*]	This study
XW8490	*yqIs100* (*Pced‐1mCherry::ACT‐1*)	(Huang et al. 2012)
JMT38	*hum‐1* (ok634) I; [*Phum‐1::gfp::hum::hum‐1 3′UTR*] *yqIs100* (*Pced‐1mCherry::ACT‐1*)	This study
JMT39	*hum‐1* (ok634) I; *hum‐5* (ok1885) III; [*Phum‐1::gfp::hum::hum‐1 3′UTR*] [*Phum5::mCherry::hum::hum‐5 3′UTR*]	This study
JMT40	[*Phum5::mCherry::hum::hum‐5 3′UTR*]	This study

### 2.5 Brood Size Assays

Single L4 worms were transferred onto individual NGM plates seeded with a small drop of *E. coli* OP50 and transferred to fresh plates every 24 h. Plates were incubated at 20°C for 48 h to distinguish between sterile and fertile eggs. Hatched juveniles were counted as offspring, while infertile eggs were excluded from brood size counts.

### 2.6 Lifespan Assays

Lifespan assays were performed at 20°C. L4 larvae were transferred onto NGM plates seeded with *E. coli* OP50, containing 50 μM FUDR. Animals were scored dead or alive every other day by gently prodding them with a platinum wire. Animals that died due to crawling up the wall of the petri dish or internal hatching of larvae were censored.

### 2.7 Fission Yeast Cell Culture

Prototrophic *Schizosaccharomyces pombe* cells were cultured, transformed, and maintained according to Moreno et al. (1991) using Edinburgh minimal medium with glutamic acid nitrogen source (EMMG). Cells were cultured at 25°C and maintained as early to mid‐log phase cultures for 48 h prior to analysis. TCA buffer cell extracts were prepared as described previously (Baker et al. 2019) and analyzed using anti‐Myo1 antibody (Attanapola et al. 2009) at 1:1000 dilution. *S. pombe* strains used in the study are listed in Table 2.

**TABLE 2:** *S. pombe* strains used in this study.

#	Genotype	Source
DM1876	Prototroph wild type	Lab stock
DM2150	*leu1.32*	Lab stock
JP1379	*ste20::kanMX6*	(Baker et al. 2016)
DM2322	*myo1::kanMX6*	Lab stock
DM829	*myo1::kanMX6 ura4.d18*	This study
DM2209	*yfp‐myo1:kanMX6*	This study
DM2360	*yfp‐myo1:kanMX6 ste20::kanMX6*	This study
DM2614	*mNeongreen‐myo1:URA4 ura4.d18*	(Baker et al. 2019)
DM2633	*mNeongreen‐myo1:URA4 sid4.tdTomato:hphMX6 ura4.d18*	(Baker et al. 2019)
DM2750	*mNeongreen‐myo1‐S782A:URA4 ura4.d18*	This study

### 2.8 Microscopy

Yeast cells were mounted onto coverslips with lectin as described previously (Baker et al. 2019). Worms were anaesthetised and mounted onto No. 1.5 coverslips under < 1 mm thick circular agarose (2%) pads, and attached with appropriate spacers onto glass slides, before being visualised on an inverted microscope (Mulvihill 2017). All live cell imaging for each sample was completed within 30 min of mounting cells onto coverslips. **Widefield**: Samples were visualised using an Olympus IX71 microscope with a UApo N 100× NA1.49 oil immersion objective lens mounted on a PIFOC z‐axis focus drive (Physik Instrumente, Karlsruhe, Germany), and illuminated using LED light sources (Cairn Research Ltd., Faversham, UK) with appropriate filters (Chroma, Bellows Falls, VT, USA). Samples were visualised using either QuantEM (Photometrics, Tucson, AZ, USA) EMCCD or Zyla (Andor, Belfast, UK) sCMOS cameras, and the system was controlled with MetaMorph software (Molecular Devices, San Jose, CA, USA). Each 3D maximum intensity projection of volume data was calculated from 21 z‐plane images, each 0.2 μm apart. **Confocal laser scanning microscopy (CLSM)**: A Nikon A1Si spectral detector confocal system was used with a plan‐apochromatic VC 60x NA 1.4 oil immersion objective (Nikon Corp., Tokyo, Japan). Images were acquired in three channels, using one‐way sequential line scans: Alexa Fluor 488 (GFP) was excited at 488 nm and its emission collected at 525/50 nm. Alexa Fluor 555 (mCherry) signal was excited at 561 nm and collected at 595/50 nm. Differential interference contrast images were acquired using the transmitted light detector. No offset was applied, and the scan speed was ¼ frames/sec (galvano scanner). Axial step size was 140 nm, with 30–50 image planes per z‐stack. **Structured‐Illumination‐Microscopy (SIM)**: Imaging was undertaken using a Zeiss Elyra PS. One microscope with a 100× NA 1.46 oil immersion objective lens (α Plan‐Apochromat, Zeiss, Oberkochen, Germany) as described previously (Eastwood et al. 2023). Briefly, cells as described above were placed onto high precision No. 1.5 coverslips (Zeiss, Jenna, Germany). A 488 nm laser was used to illuminate GFP fusions. The optical filter set consisted of a multi‐band dichroic mirror MBS 405/488/561, and a dual‐band emission filter LBF‐488/561. A total of three rotations of the structured illumination pattern were implemented to obtain two‐dimensional super‐resolution imaging information. Super‐resolution SIM image processing was performed using the Zen software (Zeiss, Oberkochen, Germany). Pearson correlation coefficient (*r*) of colocalisation between proteins was calculated using ImageJ, and *r* values denoted in the figure legends.

### 2.9 Purification of Recombinant TH1 Fusions and Lipid Binding Assay

The pET151‐D‐mNeongreen‐TH1 constructs were introduced into BL21‐DE3 *E. coli* , and 6 × HIS tagged TH1 fusion proteins were expressed for 3 h (T7 expression induced with 100 μg/mL IPTG at OD_600_ 0.5) and affinity‐purified on nickel‐agarose columns. PIP strips (Thermofisher) were blocked overnight at 4°C with PBST +3% milk, then probed with anti‐mNeongreen primary and alkaline phosphatase secondary antibodies (with appropriate washes in PBST) prior to immunodetection with BCIP‐NBT (Merck). Blots were subsequently analysed by densitometry using ImageJ software. Briefly, mean grayscale signals within 80 pixel diameter circle areas (covered each blot) were determined for each lipid. Equivalent circles from background regions were averaged and deducted from the sample values to obtain background corrected mean values (shown in Figure S2).

## 3 Results

### 3.1 Myo1d and Myo1e Localises to Discrete Groups of Cells in the Worm

The genome of *C. elegans* contains genes encoding 7 non‐conventional cytoplasmic myosins, including two class I myosins—a myosin 1d, HUM‐5, and a myosin 1e, HUM‐1 (Figure S1; Baker and Titus 1997; Johnson et al. 2022). These two long‐tailed monomeric subclasses of myosin‐1, found in organisms as diverse as slime moulds to mammals, each associate with cellular membranes, but have distinct functions, regulations, and motor activities (Pernier and Schauer 2022; McIntosh and Ostap 2016).

**FIGURE 1:**
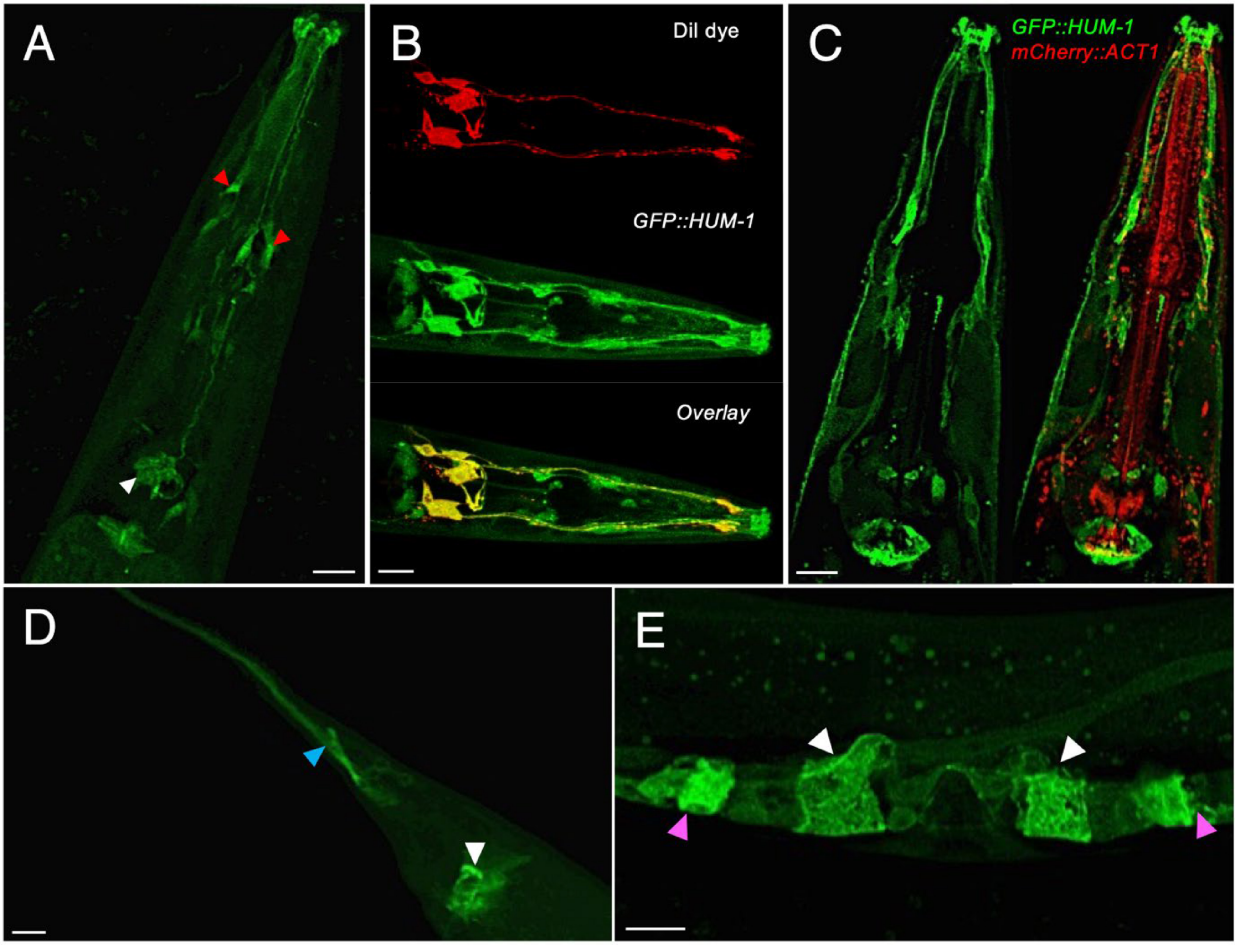
HUM‐1/Myo1e associates with discrete neuronal and gamete structures in *C. elegans* . (A) GFP::HUM‐1 (green) concentrates to the amphid sensilla (red arrowhead), inner labial sensilla (white arrowhead), pharyngeal‐intestinal valve. (B) DiI dye‐filling of the amphid neurons (red) shows co‐localisation (*r*: 0.41) with GFP::HUM‐1 (green), confirming HUM‐1 expression in these structures. (C) GFP::HUM‐1 (green) shows no co‐localisation with mCHERRY::ACT‐1 (red) (*r*: −0.02) in any of the chemo/odour sensory sensilla or the pharyngeal‐intestinal valve within the head. (D) Localisation of GFP::HUM‐1 within the tail concentrated to the phasmid sensilla and PHC neuron (blue arrowhead) and intestinal‐rectal valve (white arrowheads). (E) Within the reproductive system, GFP::HUM‐1 showed localisation to the; spermatheca/SU valve (magenta arrowhead) and uterine toroidal epithelial cells (white arrowhead). Scale bar—10 μm.

To explore the expression patterns of *C. elegans* myosin I proteins and gain insight into the cell types that they support, we generated fluorescently labelled variants of each protein, *gfp::hum1* and *mCherry::hum‐5*, and stably expressed them in *hum‐1* and *hum‐5* mutants, respectively. Visualisation of their expression pattern and cellular localisation in live, late larval (L4) stage *C. elegans* showed GFP::HUM‐1 expression concentrated within the chemo/odour sensory sensilla (specialized structures that detect environmental stimuli such as chemicals, temperature, humidity, or mechanical forces), the pharyngeal‐intestinal and intestinal‐rectal muscular valves, and the reproductive system. Specifically, within the chemo/odour sensory sensilla, GFP::HUM‐1 was seen throughout cell bodies, axons, and other processes of amphid neurons and inner labial neurons located in the head (Figure 1A). The location of amphid and phasmid neurons in the head and tail of the worms was confirmed by DiI staining (Tong and Bürglin 2010) (Figure 1B,D,E). This indicates that HUM‐1/Myo1e is implicated in the cytoskeletal networks in specific neuronal and muscle cells. To explore the localisation of HUM‐1 in relation to another key cytoskeletal protein, actin, the *hum‐1;gfp::hum‐1* reporter strain was crossed with a strain exogenously expressing tagged actin, *mCherry::act‐1* (Huang et al. 2012). Primarily expressed in muscle and myofilament‐containing cells, ACT‐1 localisation includes the isotropic bands of the body wall muscle sarcomeres, phagocytes, spermatheca/Spermatheca Uterine (SU) valve, and the pharyngeal muscle (Dixon and Roy 2005; Mango 2007; Huang et al. 2012; Wirshing and Cram 2017). However, GFP::HUM‐1 did not co‐localise with ACT‐1 in any of these tissues (Figure 1C), indicating that the *C. elegans* myosin 1e interacts with one of the other actin isoforms within these cells.

**FIGURE 2:**
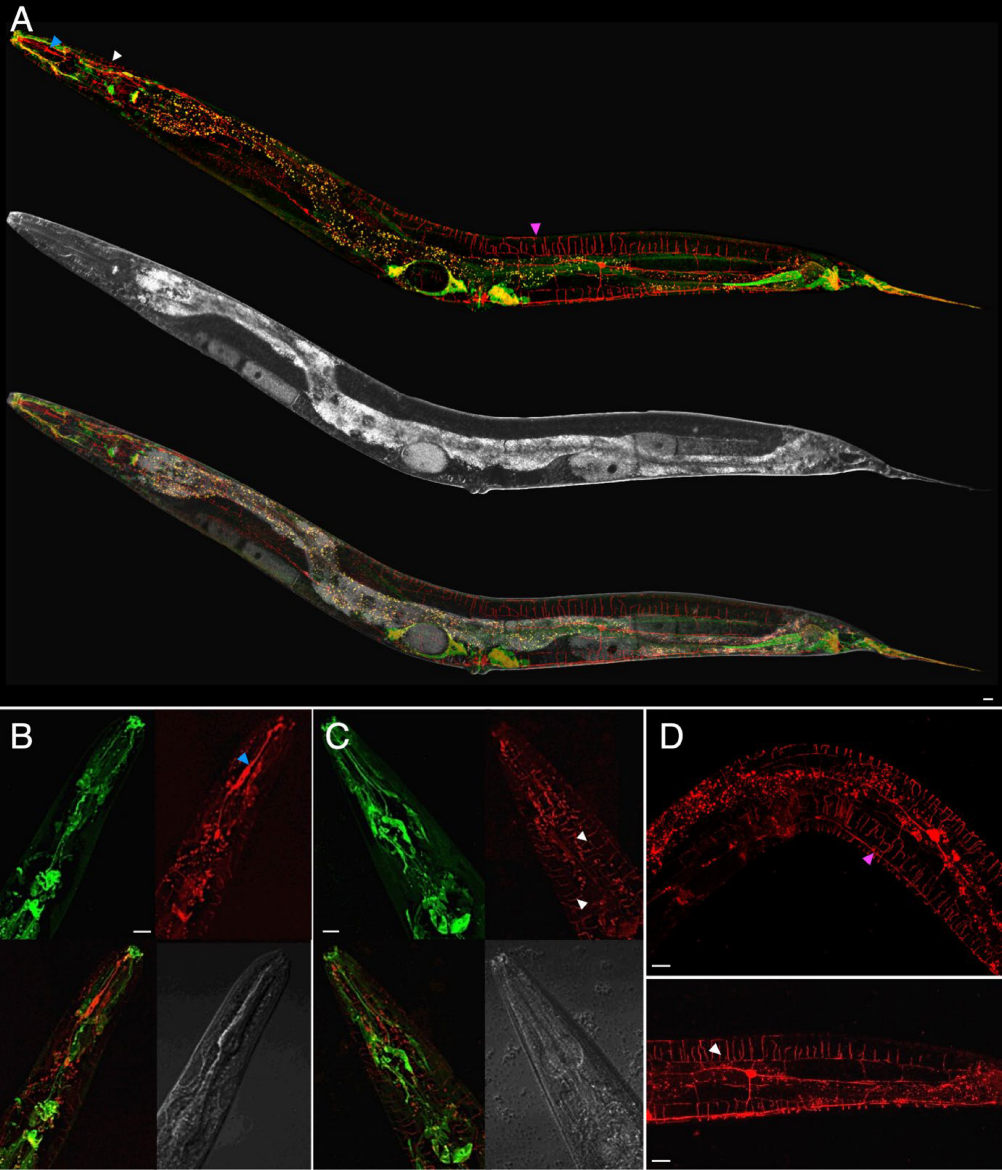
HUM‐5/Myo1d is expressed in CEP, FLP and PVD neurones of *C. elegans* . (A) Tiled confocal micrograph image of *gfp::hum1* and *mCherry::hum‐5* expressing worms reveal GFP‐HUM‐1/Myo1e (green) and mCHERRY‐HUM‐5 (red) localise to discrete separate (*r*: −0.03) sets of neurones within the adult worm. Fluorescence (upper) and transmitted (middle) signals are merged in the composite (lower image). (B) mCHERRY::HUM‐5 (red) appears to localise to CEP mechano‐sensory neurons within the nose tip (blue arrowhead); (C) FLP mechano‐sensory neurons within the head (white arrowheads); as well as (D) PVD mechano‐sensory neurons within the body wall (magenta arrowhead) of adult worms. Scale bar—10 μm.

To assess whether the two *C. elegans* class 1 myosins co‐localise, we generated animals expressing both the *gfp::hum1* and *mCherry::hum‐5* transgenes using classical genetics. However, fluorescent microscopy revealed that although mCHERRY::HUM‐5 was also localised in neurons, these were distinct from those expressing GFP::HUM‐1 (Figure 2). Specifically, we noted that mCHERRY::HUM‐5 is highly expressed in the Cephalic (CEP), FLP, and PVD neurons (www.wormatlas.org) where it localizes throughout cell bodies, axons, and other processes (Figure 2A–C). Each of these cells is necessary for mechanosensing in worms and suggests that HUM‐5/Myo1d is involved in communicating changes in tension at the membrane of these cells, which is consistent with Myo1d function in mammals in tension sensing (Dürrwang et al. 2006; Adamek et al. 2008; Laakso et al. 2008).

### 3.2 HUM‐1/Myo1e and HUM‐5/Myo1d Have a Complementary Function in Affecting Lifespan

**FIGURE 3:**
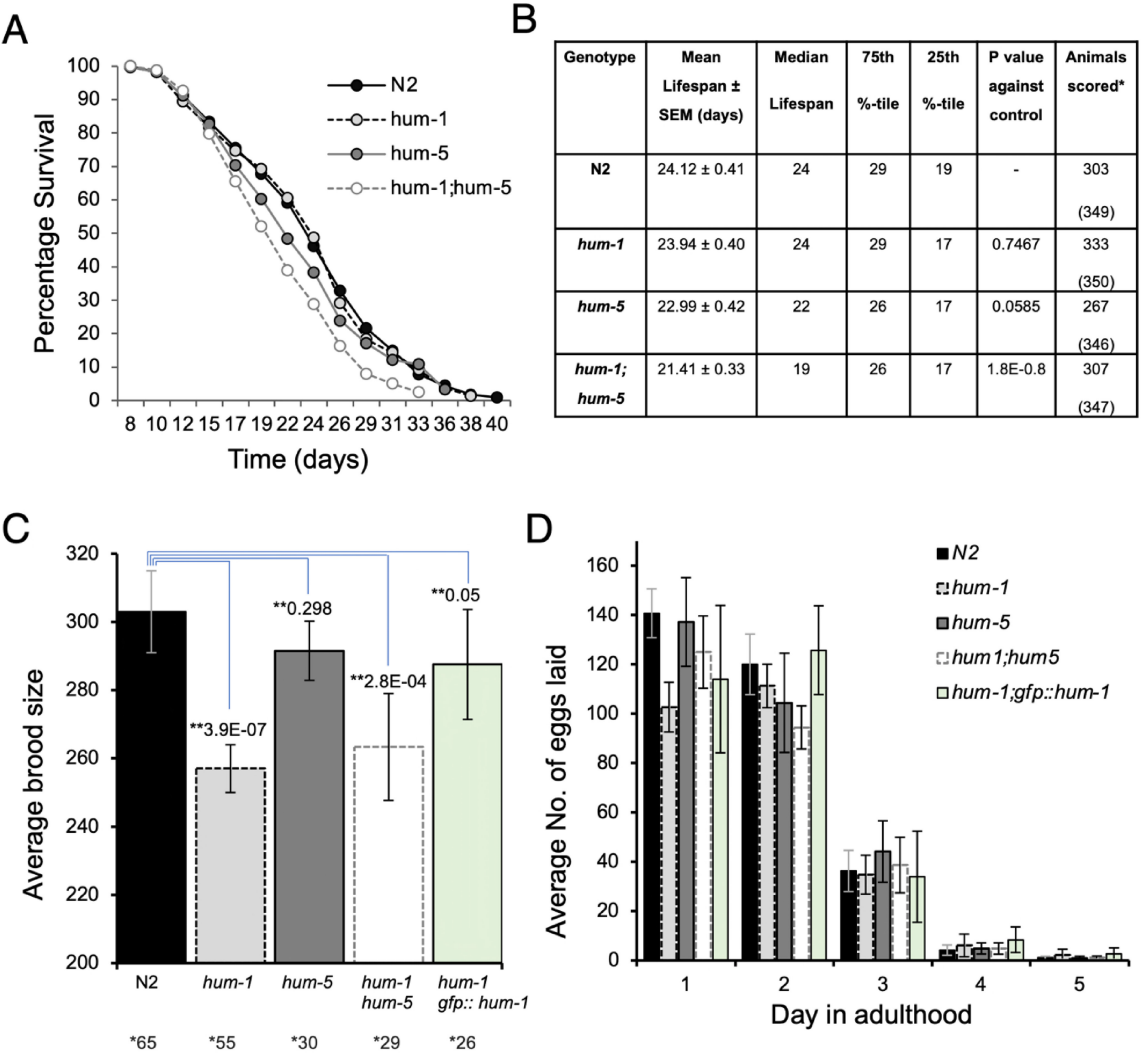
Functional analysis of *C. elegans* Myo1d and Myo1e. (A) Survival curve of N2 and myosin I mutant hermaphrodites at 20°C. N2 controls showed a mean lifespan of 24 days. Disruption of the hum‐1 or hum‐5 gene alone did appear to significantly impact lifespan. Disruption of both hum‐1 and hum‐5, however, decreased the mean lifespan to 21 days. (B) Lifespan assay data for N2 and myosin I deletion strains. SEM: standard error of the mean. 25th and 75th percentile refer to the day at which 25% or 75% of the population is dead. Animals scored*; number of animals dying senescent deaths (starting sample, including animals dying non‐senescent deaths). When compared to the N2 control, *hum‐1* or *hum‐5* mutants alone, the double mutant was significantly different with > 99.99% level of confidence (*t*‐test). (C) Disruption of *hum‐1* showed a significant reduction in average brood size, while disruption of *hum‐5* alone did not Introduction of the *gfp::hum‐1* transgene rescued the reduced brood phenotype. *Number of maternal hermaphrodites used. **t‐test value calculated against N2 control. (D) N2 control, *hum‐5*, and *hum‐1;hum‐5* disruptions showed the highest number of progeny laid on day 1 of adulthood, with this number decreasing with each consecutive day thereafter. Hermaphrodites with disruption of *hum‐1* showed a delay in embryo release, with each strain showing the highest number of progeny laid on day 2.

Given their differential expression pattern, we explored the functional roles of *C. elegans hum‐5/Myo1d* and *hum‐1/Myo1e* in a series of assays assessing nematode health. First, we compared the overall lifespan of *hum‐1* and *hum‐5* deletion mutants with that of wild‐type controls (Table 1). We found that disruption of either *hum‐1* or *hum‐5* alone did not have a statistically significant impact on nematode lifespan, with median lifespans of 24 and 23 days, respectively, compared to 24 days for the control (Figure 3A,B). However, a double mutant of both myosin I genes significantly reduced the mean lifespan to 21 days (*p* value—1.8E‐0.8 Log Rank Test). Similarly, differences between each single mutant compared with the hum‐1; hum‐5 double mutant were significantly different, with a > 99.99% level of confidence. These results indicate redundancy between *hum‐5*/Myo1d and *hum‐1*/Myo1e in maintaining the lifespan of *C. elegans* .

### 3.3 *hum‐1/*Myo1e Is Required for Maintenance of *C. elegans* Progeny Production

As HUM‐1/Myo1 was seen to localise to cells involved in reproduction (Figure 1C), we next tested the impact of *hum‐1* and *hum‐5* on progeny production. Disruption of *hum‐1* alone caused a significant reduction in brood size with an overall average of 257 ± 7, compared to 303 ± 12 for WT controls, *p* = 3.9 × 10E‐07 two‐tailed *t*‐test (Figure 3C). In contrast, disruption of *hum‐5* alone had very little impact on brood size, giving an average of 292 ± 9, *p* = 0.298. Disruption of both *hum‐1* and *hum‐5* together showed a significant decrease in brood size to 263 ± 16, *p* = 2.8E‐4 versus WT control. These results show that HUM‐1/Myo1e specifically required for *C. elegans* progeny production. To further investigate whether the reduction in brood size was *hum‐1* dependent, we rescued *hum‐1* mutants with our *gfp::hum‐1* transgene and quantified progeny production. We found that introduction of the *gfp::hum‐1* transgene rescued the brood phenotype, increasing the average brood size from 257 ± 7 to 288 ± 16, *p* = > 0.05 versus WT control. This confirms that HUM‐1 is required for normal progeny production and indicates that the *gfp::hum‐1* transgene product is functional in vivo (Figure 3C).

To explore whether *hum‐1*/Myo1e or *hum‐5*/Myo1d function affects the rate of progeny production over the *C. elegans* reproductive period, we compared the average number of progeny laid by *hum‐1* or *hum‐5* mutants per day to WT controls. Since hermaphrodites produce only a fixed number of sperm, meiotic maturation rates and embryo laying are initially highest on the first day of adulthood but decline as sperm are used for fertilization. Age‐specific fecundity is therefore positively correlated with fertility on contiguous days (Brooks and Johnson 1991). The wild‐type control showed the highest average number of progeny laid on day 1 of adulthood, with each consecutive day thereafter showing a linear decrease as sperm depletes. Despite the impact of the *hum‐1* mutation on overall progeny production, we did not observe any changes in age‐specific fecundity either in this strain or in *hum‐5* mutants (Figure 3D). *hum‐1 [gfp::hum‐1]* animals, however, appeared to have a delay in embryo release, with the highest number of progeny laid on day two. Following day two, fertility decreased on contiguous days. These results suggest that although the presence of the *gfp::hum‐1* transgene can partially rescue the overall brood size, it is unable to rescue the age‐specific fecundity phenotype (Figure 3D).

### 3.4 Phosphorylation of a Conserved AGC Kinase Consensus Site Within the Tail of Myo1e Regulates Membrane Association

**FIGURE 4:**
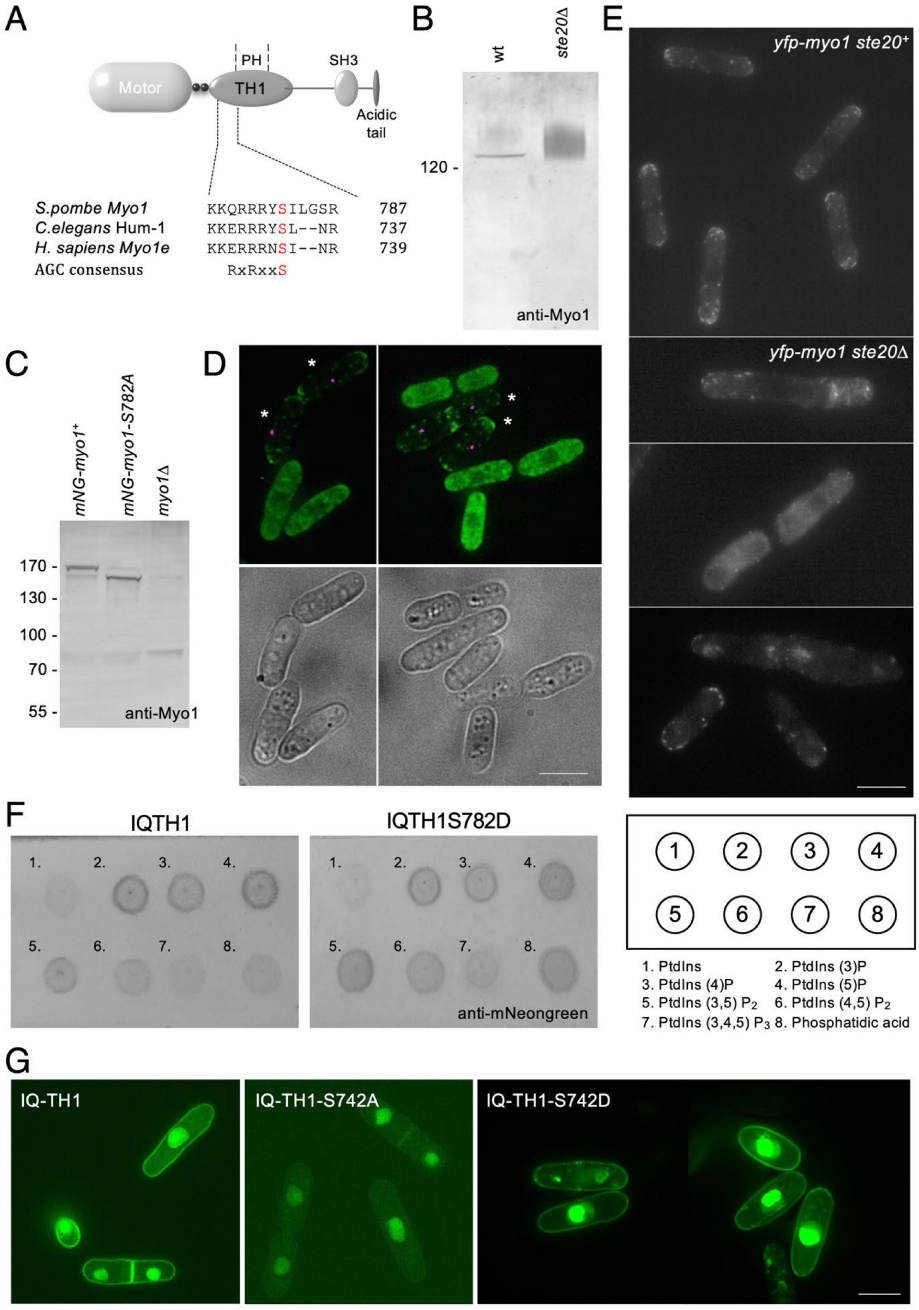
*S. pombe* Myo1 serine 782 phosphorylation regulates association with the cell membrane. (A) Schematic of myosin 1e domains and alignment of conserved phosphoserine within the TH1 domain. (B) Anti‐Myo1 western blot of extracts from wild type and *ste20∆* cells. (C) Anti‐myo1 western blot of extracts from *mNeongreen‐myo1* ^+^, *mNeongreen‐myo1‐S742A*, and parental *myo1∆* cells. (D) Micrograph of mixed *mNeongreen.myo1* ^+^ *sid4.tomato* (asterisks) and *mNeongreen.myo1.S782A* cells highlights cytoplasmic distribution of mutant protein. (E) YFP‐Myo1 fluorescence in wild type (*ste20* ^+^) and TORC2 signalling deletion (*ste20∆*) cells. A significant proportion of *ste20∆* cells lack normal Myo1 membrane association at sites of cell growth. (F) Anti‐mNeongreen (Thermofisher) blot of recombinant mNeongreen‐IQ‐TH1 (left) and mNeongreen‐IQ‐TH1‐S782D (middle) fusion protein bound PIP strip membrane. See Fig. S2 for densitometric quantification. (G) SIM images of wild type *S. pombe* containing episomal plasmids expressing mNeongreen fused to wild type, S782A, and S782D variants of the Myo1 IQ‐TH1 domain. Expression from the nmt41 promoter was minimised by the addition of thiamine (4 μM final concentration). Scale bar—10 μm.

Upon analysis of a myosin 1d and 1e sequence alignment, we identified a conserved AGC family kinase (Pearce et al. 2010) consensus site within the conserved TH1 domain of myosin 1e (Figure 4A and S1). This phosphoserine has been shown to be phosphorylated in organisms as diverse as fission yeast and humans (Wilson‐Grady et al. 2008; Beltrao et al. 2009; Carpy et al. 2014; Hornbeck et al. 2015). This signalling can be affected by environmental stress (Hálová et al. 2021). A Pleckstrin Homology (PH) domain is contained within the TH1 region, and is required for Myo1e association with cell membranes across systems (Mazerik et al. 2014; Chen et al. 2012). We were thus motivated to explore the impact phosphorylation of this conserved residue had upon the ability of Myo1e to localise across unicellular and metazoan organisms, using the fission yeast and *C. elegans* model systems. The TOR signalling pathway is essential for regulating cell growth in response to changes in the extracellular environment in unicellular and metazoan organisms (Laplante and Sabatini 2012; Hartmuth and Petersen 2009). Consistent with Myo1 being phosphorylated upon multiple residues (Baker et al. 2019) and some being TORC2 dependent, when extracts of cells lacking Ste20 (homologue of the TORC2 component, Rictor) were subjected to SDS‐PAGE and subsequent western blot analysis, Myo1 reproducibly migrated as a smear, indicative of multiple post‐translational modifications. This contrasts with equivalent wild type extracts, where Myo1 ran as a single discrete band, equivalent to its predicted size of ~135 kDa (Figure 4B). To explore the role TH1 domain phosphorylation has on yeast Myo1, a strain was generated in which a confirmed *myo1::kanMX6* deletion locus was replaced with an *mNeongreen‐myo1‐S782A* allele. Anti‐Myo1 western blots revealed Myo1 expression levels were comparable in extracts from prototrophic equivalent *mNeongreen‐myo1‐S782A* and *mNeongreen‐myo1* ^+^ cells (Baker et al. 2019), with differences in migration consistent with an unphosphorylated residue replacement (Figure 4C). Myo1 localisation was compared simultaneously in *mNeongreen‐myo1‐S782A* cells and *mNeongreen‐myo1* ^+^ cells also expressing a tdTomato tagged variant of the essential spindle body component Sid4 (Chang and Gould 2000). While mNeongreen‐Myo1 localised to endocytic foci at sites of cell growth (Lee et al. 2000; Sirotkin et al. 2005; Attanapola et al. 2009), mNeongreen‐Myo1‐S782A failed to associate with membranes and instead had a cytoplasmic distribution within the yeast cell (Figure 4D). This is consistent with the cytoplasmic Myo1 localisation observed in a significant proportion of cells lacking the conserved regulatory component of TORC2, Rictor^Ste20^ (Figure 4E). The *myo1‐S782A* cells displayed normal polarised growth morphology, which contrasts with the *myo1::kanMX6* deletion parental cells from which the strain was generated, which, as previously reported, exhibited significant growth and polarity defect phenotypes (Baker et al. 2019). These data indicate phosphorylation at this conserved residue is required for Myo1 to associate normally with the plasma membrane, but is not required for its role in maintaining polarised cell growth.

While we failed to generate an equivalent correctly integrated phospho‐mimetic *myo1‐S782D/E* allele, we examined the impact phosphorylation had upon the direct membrane binding capacity of the Myo1‐TH1 domain. Wild‐type and Myo1‐S782D variants of mNeongreen‐IQ‐TH1 domain fusion proteins were expressed and purified from *E. coli* and used to probe PIP strips to examine interactions between the TH1 domain fusions and different phosphoinositide head groups (Figure 4F). Anti‐mNeongreen western blot analysis (Figure 4F) and associated densitometry analysis (Figure S2) revealed that replacing serine 782 with a phosphomimetic aspartic acid residue significantly enhanced the affinity of the TH1 domain for the anionic diphospho‐ and triphospho‐PtdIns phospholipids (‐PtdIns (3,5) P_2_, ‐PtdIns (4,5) P_2_, ‐PtdIns (3,4,5) P_3_) as well as phosphatidic acid, each of which has significant differences in surface charge and plays key roles in cell signalling.

Equivalent GFP‐IQ‐TH1 fusion proteins were subsequently expressed from appropriate episomal plasmids in wild‐type fission yeast cells to examine the ability of the proteins to associate with membranes in vivo (Figure 4G). Consistent with previous studies, Structured Illumination Microscopy (SIM) revealed the wild‐type IQ‐TH1 domain fusion clearly associated with the plasma membrane and nuclear envelope (Lee et al. 2000). An equivalent non‐phosphorylatable TH1‐S782A fusion displayed a reduced association with membranes within live yeast cells, with a much smaller proportion of the protein associating with the plasma membrane (Figure 4G). In contrast, the phospho‐mimetic TH1‐S782D fusion variant not only localised tightly with the plasma membrane but was frequently seen to associate with cytoplasmic vesicles as well as nuclear membranes displaying an aberrant morphology (Figure 4G). Thus, phosphorylation of the conserved Myo1 TH1 domain serine782 regulates the ability of this Myo1e motor to associate with membranes according to their specific phospholipid composition.

**FIGURE 5:**
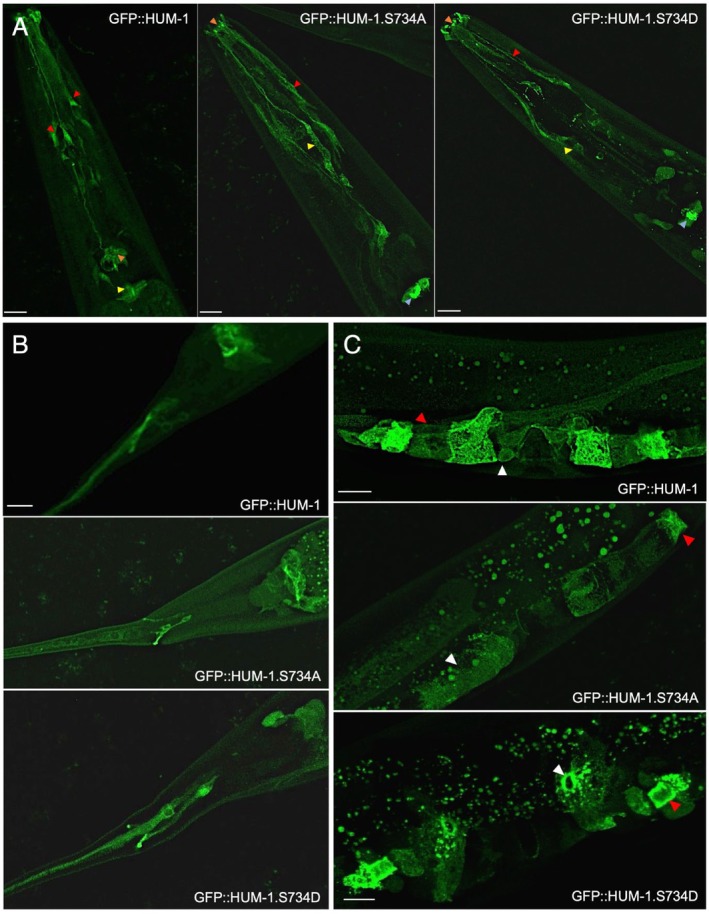
Modulation of *C. elegans* HUM‐1/Myo1e serine 734 phosphorylation status affects interactions with membranes. (A) Expression of GFP::HUM‐1 is seen throughout cell bodies, axons and other processes of the amphid sensilla (red arrowheads) and inner labial sensilla (orange arrowheads) and the pharyngeal‐intestinal valve (yellow arrowheads). In contrast, neurons expressing GFP::HUM‐1S734A have elongated commissures (yellow arrowheads) with less uniform distribution of axons (red arrowheads). Localisation appears more cytoplasmic within axons and commissures, with reduced discrete localisation in the lips (orange arrowheads). Expression in the phalangeal—intestinal valve is more concentrated to the inner membrane (blue arrowheads). GFP::HUM‐1S734D shows less cytoplasmic and more punctate localisation in the neuronal dendrites (red arrowheads). Neuron commissures are not as well defined (yellow arrowheads). Expression within the lips appears more concentrated to the neuronal tips (orange arrowheads). More discrete localisation can be seen on the inner membrane of the phalangeal‐intestinal valve (blue). (B) GFP::HUM‐1S734A and S734D show comparable expression to GFP::HUM‐1 in the intestinal‐rectal valve and phasmid sensilla however, S734A shows reduced expression in the PHC neuron. Scale bar is 10 μm (C) GFP::HUM‐1S734A shows more cytoplasmic localisation in ut cells (white arrowheads) with less expression in the spermatheca and SU valve (red arrowheads). GFP::HUM‐1S734D shows comparable expression to GFP::HUM‐1 in the spermatheca and SU valve (red) however appears to localise more to the membrane of ut1 (white arrowheads). Wild type GFP::HUM‐1 images from Figure 2 repeated in (B) and (C) to ease direct comparison for reader. Scale bar—10 μm.

Finally, we examined how replacing the equivalent conserved serine within *C. elegans* HUM‐1/*Myo1e*TH1 domain impacted the ability of the full‐length protein to localise and function within the worm. Separate constructs containing *gfp::hum‐1‐S734A* and *gfp::hum‐1‐S734D* were generated (see methods), introduced into *hum‐1* null animals, and the distribution of the subsequent proteins examined (Figure 5). In all cells examined, the GFP::HUM‐1S734D protein had a brighter, more distinct, and tighter membrane‐associated localisation pattern when compared to the wild‐type protein, while the non‐phosphorylatable GFP::HUM‐1S734A protein was more diffuse and cytoplasmic within the cells. Interestingly, neither HUM‐1 phosphomutants, S734A or S734D, were able to rescue the *hum‐1* mutant brood size or fecundity phenotypes (data not shown), which suggests that dynamic phosphoregulation of this conserved residue may be critical for the proper regulation of membrane association and function of this myosin motor protein.

## 4 Discussion

Here we describe an analysis of the localisation and function of the *C. elegans* class I myosins, HUM‐5/Myo1d and HUM‐1/Myo1e, and establish that phosphorylation of a conserved serine within the Myo1e TH1 domain regulates the association of the motor protein within the cell membrane in both *C. elegans* and the fission yeast, *S. pombe*. We found HUM‐1/Myo1e is expressed and localises to membranes within the chemo/odour sensory sensilla (Figure 1), while HUM‐5/Myo1d is seen to localise exclusively to the CEP, FLP, and PVD mechanosensory neurons (Figure 2). While we were unable to confirm these localisations by immunofluorescence due to lack of specific antibodies, these distributions are consistent with the auditory and mechanosensory functions that have been defined for Myo1d motors in animals and suggest a role in sensing in the worm. In *C. elegans* , mechanosensory neurons are responsible for diverse sensory modalities including responses to low‐threshold mechanical stimuli, temperature, propioception, and nociception (Albeg et al. 2011). The PVD mediates the response to high‐threshold mechanical stimuli and thus functions as a nociceptor, while FLP neurones are involved in harsh nose touch and gentle nose touch responses to activate an escape behaviour. Given these localisation patterns, we were surprised to observe that removing either (or both) class I myosins had no impact upon (i) the diacetyl chemotaxis index or (ii) the harsh and gentle mechanosensing of the nose or posterior of the worms (data not shown). However, a consistent and significant reduction in lifespan was observed in animals lacking both *hum‐5*/Myo1d and *hum‐1*/Myo1e (Figure 3). The fact that this lifespan phenotype is only observed in the double mutant could be due to redundancy between *hum‐1* and *hum‐5*. These genes may be acting subtly in their different tissues, with their combined absence having a greater effect, or may be implicated in tissue‐tissue signalling that controls overall lifespan. While it is possible the myosin I localised within these neurons may play a subtle, as yet undefined role in the development of the worm, it is likely that the combined impact of deleting both class I myosins has an additive negative impact upon the growth and development of cells across the organism. In addition to the chemo/odour sensory sensilla neurones, GFP‐tagged HUM‐1/Myo1e was also observed within the pharyngeal‐intestinal and intestinal‐rectal valves and the reproductive system (Figure 1). Localisation within these groups of cells is consistent with the observed impact that the *hum‐1* disruption has upon maximum brood size, timing of embryo release, and age‐specific fecundity of worms (Figure 3). Together, these data give a strong indication that Myo1e plays an important role in regulating the formation and organisation of neurones and Spermatheca/SU within the worm.

We went on to establish how phosphorylation of a conserved phosphoserine within the TH1 domain of Myo1e affected the localisation and function of the motor protein in *C. elegans* as well as the simple unicellular fission yeast, *S. pombe*. In both cases, replacing the serine with a non‐phosphorylatable alanine residue resulted in disruption of the Myo1e membrane interaction (Figures 4 and 5). Interestingly, while replacement with a phosphomimetic aspartic acid resulted in a tighter association with membranes in worms, this mutant was unable to complement *hum‐1* function (not shown). Consistent with this result, expression of a IQ‐TH1‐S782D fusion in fission yeast resulted in localisation to additional membranes and disruption of nuclear morphology (Figure 4). These data indicate phosphorylation of this conserved residue is required to modulate the interaction with specific membranes within the cell. Interestingly, like Myo1, the TORC2 complex associates with the plasma membrane (Baker et al. 2016) at sites of cell growth, so it is at the appropriate location to stimulate Myo1 membrane interactions when required. Consistent with this localised activation model, the majority of Myo1 remains cytoplasmic in a significant proportion of cells lacking TORC2 (Figure 4), suggesting this signal pathway is at least in part responsible for regulating endocytosis and growth through modulating association with specific lipid membranes in response to both cell cycle and environmental stimuli.

This study illustrates the critical role TH1 phosphorylation plays in regulating Myo1e membrane association at specific locations. It also provides a potential method to modulate and facilitate myosin release from PM after endosome release, to facilitate myosin I dependent membrane reorganisation and coordinated regulation of cell growth. In fission yeast, overexpression of full length Myo1 drives association with the entire plasma membrane and stimulates a dramatic reorganisation of the membrane, resulting in aberrant septum formation (Attanapola et al. 2009). Similarly, mutant Myo1 that fails to associate with actin also interacts indiscriminately with the plasma membrane (Attanapola et al. 2009), but like here, displays normal polarised cell growth. Thus, full length, active Myo1e is required for correct localisation with the membrane. It is now apparent that not only the intrinsic biophysical properties of the Myo1e motor protein are necessary for its correct cellular localisation but interactions with additional other proteins are also required. These include calmodulin light chains (Baker et al. 2019), as well as additional regulators such as the fission yeast ankyrin repeat protein, Ank1, which interacts with Myo1 via its TH1 domain to target it to sites of endocytosis (Willet et al. 2023).

It is intriguing to consider the potential cooperative nature of different phosphorylation events, such as the IQ domain site (S742), which impacts light chain binding and neck conformation (Baker et al. 2019), and the TH1 site (S782), as described here, on the fission yeast Myo1e. The combined impact of the phosphorylation status of these two residues, for example, would have a significant impact on both light chain association and conformation of the motor protein. Thus, further research and analysis are required to explore how the combined impact of multiple phosphorylation events impacts the overall regulation and subsequent function across the different classes of myosin.

## Author Contributions

H.R.B. and K.B. performed the experimental studies; M.E. performed *C. elegans* microinjections; D.P.M., P.P.L., and L.W. supervised widefield, confocal, and SIM microscopy respectively; J.M.T. and D.P.M. supervised research; D.P.M. and M.A.G. sought funding and managed the overall project. All authors designed experiments. D.P.M. wrote main drafts of the manuscript, and all authors contributed to editing.

## Conflicts of Interest

The authors declare no conflicts of interest.

## Supporting information


**Data S1.** Supporting Information.

## Data Availability

The data that support the findings of this study are available on request from the corresponding author. The data are not publicly available due to privacy or ethical restrictions.
