# Supplementary material for: Conserved Phosphorylation of the Myosin1e TH1 Domain Impacts Membrane Association and Function in Yeast and Worms
Source: Cytoskeleton (Hoboken). 2025 Apr 9;82(12):837–49. doi: 10.1002/cm.22026 (PMC12701525; doi:10.1002/cm.22026)

Supplementary Figure 1: (a) Phylogenetic tree from Clustal Omega alignments of Human and *C. elegans* class 1 myosins. (b) Clustal Omega alignments of class 1d & 1e myosins from *S. pombe, C. elegans,* and humans.

(a)

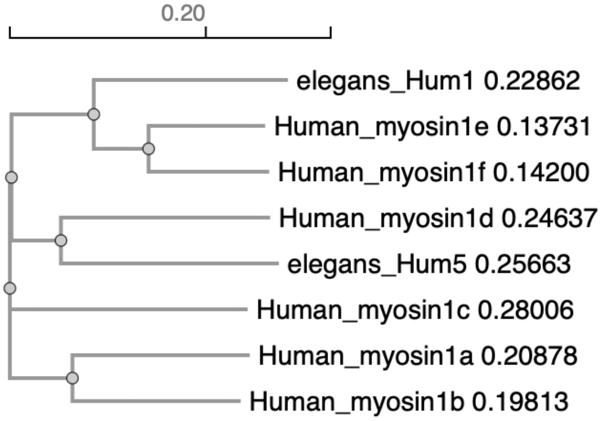

(b) *Myo1e TH1 domain highlighted in grey. Conserved TH1 phosphoserine bold red text.*

```
                                                  Motor domain
C.elegans Hum-5     -----------------------------MSYG-GHDPNGYGVEDLVLLSTIDLKSVVQN 30
H.sapiens Myo1d     -------------------------------MA-EQESLEFGKADFVLMDTVSMPEFMAN 28
S.pombe Myo1        MAILKRTNRAKAATAAAPNSTGKSNGIKKAVYT-STRKKTVGVDDLTLLSKITDEEINKN 59
C.elegans Hum-1     ---------------------------MAFHWQSKVNVQHVGVDDMVLLPKLTEQSIVEN 33
H.sapiens Myo1e     ---------------------MGSKGVYQYHWQSH-NVKHSGVDDMVLLSKITENSIVEN 38
                                                                 *  *:.*: .:   ..  *
                                                  Motor domain
C.elegans Hum-5     LQLRFQKGRIYTYIGEVLVAVNPYRQLGIYEKSTVDQYKGREIYERAPHVFAIADAAYRS 90
H.sapiens Myo1d     LRLRFEKGRIYTFIGEVVVSVNPYKLLNIYGRDTIEQYKGRELYERPPHLFAIADAAYKA 88
S.pombe Myo1        LELRFRNGEIYTYIGHVLISVNPFRDLGIYTMDILKSYQGKNRLETSPHVYAIAENAYYQ 119
C.elegans Hum-1     LKKRLQANSIFTYIGPVLISVNPFKQMPYFTEKEMLLYQGAAQYENAPHIYALADNMYRN 93
H.sapiens Myo1e     LKKRYMDDYIFTYIGSVLISVNPFKQMPYFGEKEIEMYQGAAQYENPPHIYALADNMYRN 98
                    *. *   . *:*:** *:::***:: :  :  . :  *:*    *  **::*:*:  *
                                                  Motor domain
C.elegans Hum-5     MKRFGRDSCIVISGESGAGKTETSKIIMKYLAAITNVRQQGEIERVKNVLLRSNCILEAF 150
H.sapiens Myo1d     MKRRSKDTCIVISGESGAGKTEASKYIMQYIAAITNPSQRAEVERVKNMLLKSNCVLEAF 148
S.pombe Myo1        MKSYHENQCIIISGESGAGKTEAAKRIMQYITHVSKS-VGTEIERVSEIILATNPLLESF 178
C.elegans Hum-1     MLIDNESQCVIISGESGAGKTVNAKFIMNYISRISG--GGQKVQHIKDVILQSNPLLEAF 151
H.sapiens Myo1e     MIIDRENQCVIISGESGAGKTVAAKYIMSYISRVSG--GGTKVQHVKDIILQSNPLLEAF 156
                    *    .. *::**********  :* **.*:: ::      ::::.:::*  :* :**:*
                                                  Motor domain
C.elegans Hum-5     GCAKTTRNDNSSRFGKYMHINFDYDGDPVGGNISNYLLEKSRVVRQQEGERNFHVFYQLV 210
H.sapiens Myo1d     GNAKTNRNDNSSRFGKYMDINFDFKGDPIGGHINNYLLEKSRVIVQQPGERSFHSFYQLL 208
S.pombe Myo1        GCAKTLRNNNSSRHGKYLEMIFNSGGVPVGAKITNYLLEKNRIVNQVRNERNFHIFYQFT 238
C.elegans Hum-1     GNSATVRNWNSSRFGKYVEIVFSRGGEPIGGKLSNFLLEKSRVVHQNEGDRNFHVFYQLC 211
H.sapiens Myo1e     GNAKTVRNNNSSRFGKYFEIQFSPGGEPDGGKISNFLLEKSRVVMRNPGERSFHIFYQLI 216
                    * : * ** ****.***..: *.  * * *.::.*:****.*:: :  .:*.** ***:
                                                  Motor domain
C.elegans Hum-5     NGGDDGLLRQFGLTKDAKQYYFLNQGQSHKVASINDSRDFAEVQTALRSIHTFDKQDVES 270
H.sapiens Myo1d     QGGSEQMLRSLHLQKSLSSYNYIHVGAQLK-SSINDAAEFRVVADAMK-VIGFKPEEIQT 266
S.pombe Myo1        KSAPQKYRDTYGIQ-GPENYVYTSACQCLSVDGISDEKDFQGTMNAMK-VIGITEPEQDE 296
celeganshum1        AGADKNLRSTFGIG-ELQYYNYLNMSGVFKADDTDDGKEFESTLHAMK-VVGVNDQDQLE 269
H.sapiens Myo1e     EGASAEQKHSLGIT-SMDYYYYLSLSGSYKVDDIDDRREFQETLHAMN-VIGIFAEEQTL 274
                     ..         :    . * :       .  . .*  :*  .  *:. :  .   :
                                                  Motor domain
C.elegans Hum-5     MWSVIAGLIHLGNVRFIDGENSSGAVHIAEKAALQNAARCLNVTPDELAKSLSSQVVAA- 329
H.sapiens Myo1d     VYKILAAILHLGNLKFVVDGDTPL---IENGKVVSIIAELLSTKTDMVEKALLYRTVAT- 322
S.pombe Myo1        IFRMLSIILWLGNIQFQEGQDGGS--VISDKSITEFLGYLIGVPVAAIERALTIRIMQTQ 354
C.elegans Hum-1     VLRIVATVLHIGNITFTEENNFAA---VSGKDYLEYPAFLLGLTSADIEAKLTGRKMESK 326
H.sapiens Myo1e     VLQIVAGILHLGNISFKEVGNYAA---VESEEFLAFPAYLLGINQDRLKEKLTSRQMDSK 331
```

```
                        :  ::: :: :**: *    :      :         .  :.     :   *  : : :
                                              Motor domain
C.elegans Hum-5     ----HGDIVKKQHDVNAAYYTRDALAKALYERLFSWMVSKVNEAISVQNSS--RYSKSHV 383
H.sapiens Myo1d     ----GRDIIDKQHTEQEASYGRDAFAKAIYERLFCWIVTRINDIIEVKNYDTTIHGKNTV 378
S.pombe Myo1        HGARRGSVYEVPLNPTQALAVRDALSMAIYNCLFDWIVERVNKALVTSDN-----SVSNS 409
C.elegans Hum-1     WGTQKEE-IDMKLNVEQASYTRDAWVKAIYARLFDYLVKKVNDAMNITSQS---TSDNFS 382
H.sapiens Myo1e     WGGKSES-IHVTLNVEQACYTRDALAKALHARVFDFLVDSINKAMEK-------DHEEYN 383
                          .  .      *   ***   *::  :* ::*  :*. :            .
                                              Motor domain
C.elegans Hum-5     IGVLDIYGFEIFGTNSFEQLCINYCNEKLQQLFIELVLKQEQEEYEREGIKWVKIEYFNN 443
H.sapiens Myo1d     IGVLDIYGFEIFDNNSFEQFCINYCNEKLQQLFIQLVLKQEQEEYQREGIPWKHIDYFNN 438
S.pombe Myo1        IGILDIYGFEIFENNSFEQLCINYVNEKLQQIFIELTLKTEQEEYVREQIAWTPIKYFNN 469
C.elegans Hum-1     VGILDIYGFEIFNNNGFEQFCINFVNEKLQQIFIELTLKAEQEEYVREGIKWTEIDYFDN 442
H.sapiens Myo1e     IGVLDIYGFEIFQKNGFEQFCINFVNEKLQQIFIELTLKAEQEEYVQEGIRWTPIEYFNN 443
                    :*:********* .*.***:***: ******:**:*.** ***** :* * *  *.**:*
                                              Motor domain
C.elegans Hum-5     KVICDLVEIP--RTGILSILDEACASI---GNVTDKVFLGELDKKLKSHKHYTSRNLKQS 498
H.sapiens Myo1d     QIIVDLVEQQ--HKGIIAILDDACMNV---GKVTDEMFLEALNSKLGKHAHFSSRKLCAS 493
S.pombe Myo1        KVVCDLIESK-RPPGLFAAMNDAIATAHADSAAADSAFAQRLN-FLSSNPHFEQRQ---- 523
C.elegans Hum-1     KIVCDLIETK-RPPGIMSLLDDTCAQNHGQREGVDRQLLTTLSKSFAGHPHFGPGS---- 497
H.sapiens Myo1e     KIVCDLIENKVNPPGIMSILDDVCATMHAVGEGADQTLLQKLQMQIGSHEHFNSWN---- 499
                    ::: **:*      *::: :::.         .*  :   *.  :  : *:   .
                                              Motor domain
C.elegans Hum-5     DKSMGFE-EFKITHYAGDVTYSVMGFMDKNKDTLFQDLKRLLYHSKNRLVKSLFPDGSKS 557
H.sapiens Myo1d     DKILEFDRDFRIRHYAGDVVYSVIGFIDKNKDTLFQDFKRLMYNSSNPVLKNMWPEGKLS 553
S.pombe Myo1        -------NQFIVKHYAGDVTYSITGMTDKNKDQLATDILNLIHSSNNEFMKSIFPVAEES 576
C.elegans Hum-1     -------DSFVIKHYAGDVTYNVDGFCDRNRDVLYPDLILLMQKSSRPFIQALFPENVAA 550
H.sapiens Myo1e     -------QGFIIHHYAGKVSYDMDGFCERNRDVLFMDLIELMQSSELPFIKSLFPENLQA 552
                           * : ****.* *.: *: ::*:* *   *:   *:   *.  .:: ::*    :
                                              Motor domain
C.elegans Hum-5     MAEVNRRPPTAGFLFKNSMSELVKQLAQKEPHYIRCIKPNEEKNSNVFDLERVEHQVRYL 617
H.sapiens Myo1d     ITEVTKRPLTAATLFKNSMIALVDNLASKEPYYVRCIKPNDKKSPQIFDDERCRHQVEYL 613
S.pombe Myo1        --NSRRRPPTAGDRIKTSANDLVETLMKCQPSYIRTIKPNQTKSPNDYDQQMVLHQIKYL 634
C.elegans Hum-1     --SAGKRPTTFSTKIRTQANTLVESLMKCSPHYVRCIKPNETKRPNDWEESRVKHQVEYL 608
H.sapiens Myo1e     --DKKGRPTTAGSKIKKQANDLVSTLMKCTPHYIRCIKPNETKKPRDWEESRVKHQVEYL 610
                      .    ** *  .  ::..     **. *  .   * *:* ****: *   . :: .    **:.**
                                              Motor domain
C.elegans Hum-5     GLLENVRVRRAGFAHRMPYDRFVNRYKLICAST----WPNPRRGQQLKDSCMQILESAGL 673
H.sapiens Myo1d     GLLENVRVRRAGFAFRQTYEKFLHRYKMISEFT----WPNHDLPSD-KEAVKKLIERCGF 668
S.pombe Myo1        GLQENIRIRRAGFAYRQAFDTFAQRFAVLSGKTSYAGEYTWQ--GDDKSACEQILKDTNI 692
C.elegans Hum-1     GLRENIRVRRAGFAYRRAFDKFAQRYAIVSPQT----WPCFQ--GDQQRACEIICDSVHM 662
H.sapiens Myo1e     GLKENIRVRRAGYAYRRIFQKFLQRYAILTKAT----WPSWQ--GEEKQGVLHLLQSVNM 664
                    ** **:*:****:*.*   :: *  :*: ::    *                 : : .     :  .   :
                                  Motor domain                       Neck region
C.elegans Hum-5     AQD-CVQGRTKIFIRSPQTVFRLEELRTEQLPNVITFLQKMVRGVQQRERYRRMLAVRKI 732
H.sapiens Myo1d     QDD-VAYGKTKIFIRTPRTLFTLEELRAQMLIRIVLFLQKVWRGTLARMRYKRTKAALTI 727
S.pombe Myo1        PSSEYQMGTSKVFIKNPETLFALEDMRDKFWDTMATRIQRAWRSYVRRRSEAAA----CI 748
C.elegans Hum-1     EKNQYQMGKTKIFVKNPESLFLLEETRERKFDGYARVIQKAWRQFSARKQHI-------- 714
H.sapiens Myo1e     DSDQFQLGRSKVFIKAPESLFLLEEMRERKYDGYARVIQKSWRKFVARKKYV-------- 716
                     ..     *  :*:*:: *.::* **: *  .          :*:   *     *
                            Neck region
C.elegans Hum-5     IGAYRRYKLKSYIWQLINAFRDVRRMRDLGKSIRWPAPPLVLAQFVSRLRVMHQRWRAAT 792
H.sapiens Myo1d     IRYYRRYKVKSYIHEVARRFHGVKTMRDYGKHVKWPSPPKVLRRFEEALQTIFNRWRASQ 787
S.pombe Myo1        QKLWNRNKVN----------MELERVR--------------------------------- 765
C.elegans Hum-1     -------------------------KQK--------------------------------- 717
H.sapiens Myo1e     -------------------------QMR--------------------------------- 719
                                                :

C.elegans Hum-5     ILARMPPHLRASLPQKIAAFEVLNNKKENWG--Y--TRMWRGDYLSQQEELELPTTVSTY 848
H.sapiens Myo1d     LIKSIPASDLPQVRAKVAAVEMLKGQRADLG--L--QRAWEGNYLASKPD--TPQTSGTF 841
S.pombe Myo1        ----------------NEGTKLLQGKKQRRRYSILGSRKFYGDYLSASKPNG---T---- 802
C.elegans Hum-1     ----------------EQAADLMYGKKERRRYSL--NRNFVGDYIGLEHHPT-------- 751
H.sapiens Myo1e     ----------------EEASDLLLNKKERRRNSI--NRNFIGDYIGMEEHPE-------- 753
                                      .  .:: .::             *  :  *:*:.  .

C.elegans Hum-5     HDGIQALRQSHPFGKVLFS----TYVQKFNKFNKSSLRVLIVTDRFVAKLENKKFK---- 900
H.sapiens Myo1d     VPVANELKRKDKYMNVLFS----CHVRKVNRFSKVEDRAIFVTDRHLYKMDPTKQY---- 893
S.pombe Myo1        ---LWNTCGLSQNDHVIFSMRCEVLVHKLGRTSKPSPRQLVLTKKNLYLVITKIVDQ--- 856
```

```
C.elegans Hum-1     ---LQSLVGK--RQRVLFA----CTANKYDRKFRVTKLDLLLTVNHLTLIGKEKVKNGPE 802
H.sapiens Myo1e     ---LQQFVGK--REKIDFA----DTVTKYDRRFKGVKRDLLLTPKCLYLIGREKVKQGPD 804
                                  .: *:      . * .:  :     :.:* . :  :

C.elegans Hum-5     ------LLKEPIPLQSISRISVCAESNGLFVIHVGDN---D-IVGC--AKNTKNEERVGE 948
H.sapiens Myo1d     ------KVMKTIPLYNLTGLSVSNGKDQLVVFHTKDN---KDLIVCLFSKQPTHESRIGE 944
S.pombe Myo1        --KLTQQVEKKFAVSSIDSVGLTNLQDDWVAIRNKSSQNGDMFLRCFFK---------TE 905
C.elegans Hum-1     KGKIVEVIKRQFDLPQIKSIGLSPYQDDFVILYLG-NDDYSSLLETPFK---------TE 852
H.sapiens Myo1e     KGLVKEVLKRKIEIERILSVSLSTMQDDIFI--LH-EQEYDSLLESVFK---------TE 852
                           : . : :  :  :.:   .:  .      .   . ::               *

C.elegans Hum-5     MIGTLLAHYDKITMRRSPVLIQSAVVCTLG---------GKTRTIRVFDAENNNVPPVFK 999
H.sapiens Myo1d     LVGVLVNHFKSEK-RHLQVNVTNPVQCSLH---------GKKCTVSVETRLNQP-QPDFT 993
S.pombe Myo1        FITTLKRINRNI-----QVIVGPTIQYCRK--------PGKVQTVKTAKDETTKDYDY-- 950
C.elegans Hum-1     FCTALSKAYKERTNGTLHLDFRSSHVVSYKKMKFDF--SDGKRTVQFGNDGTSSAEKTLK 910
H.sapiens Myo1e     FLSLLAKRYEEKTQKQLPLKFSNTLELKLKKENWGPWSAGGSRQVQFHQ--GFGDLAVLK 910
                    :   *     .       : .                  .    :

C.elegans Hum-5     KNG-NDVDLICHQLTAQVA----------------------------------------- 1017
H.sapiens Myo1d     KNR-SGFILSVPGN---------------------------------------------- 1006
S.pombe Myo1        --YKSGTIHVGTGLPPTSKSKPFPRLATGGSTAAARG----PRPVVQNKPAATKPVSMPA 1004
C.elegans Hum-1     PNGKVLNVSIGTGLPNTTRPSTERPQ--GGYTPRRDQLRTSTRRTKQNNQSYGQ-NGQSQ 967
H.sapiens Myo1e     PSNKVLQVSIGPGLPKNSRPTRRNTTQNTGYSSGTQNANY-PVRAAPPPPGYHQ-NGVIR 968


C.elegans Hum-5     ------------------------------------------------------------ 1017
H.sapiens Myo1d     ------------------------------------------------------------ 1006
S.pombe Myo1        AKSKPAPMANPV-----STAQQTQNRPPAPAMQARPNTTQAAAPVTSTTTTIKQATTVSA 1059
C.elegans Hum-1     AMRAPVPAHGMNNN------Y---NQTPAPVS---TNHQYSQEPA--------------- 1000
H.sapiens Myo1e     NQYVPYPHAPGSQRSNQKSLYTSMARPPLPRQQSTSSDRVSQTPE--------------- 1013

                                                SH3 domain
C.elegans Hum-5     ------------------------------------------------------------ 1017
H.sapiens Myo1d     ------------------------------------------------------------ 1006
S.pombe Myo1        SKPAPSTVTSAASSPSNISKPSAPVANNVS-----KPSAVPPPPPPPPAEVEKKDLYLAL 1114
C.elegans Hum-1     RIPVMGNVIN------Q--LNNMNLSGNGNSPAGRGPPPARGPKPPPPAKPKLNPVVIAV 1052
H.sapiens Myo1e     SLDFL-----------K--VPDQGAAGVRRQTTSRPPPAGGRPKPQPKPKP-QVPQCKAL 1059

                                           SH3 domain
C.elegans Hum-5     ------------------------------------------------------------ 1017
H.sapiens Myo1d     ------------------------------------------------------------ 1006
S.pombe Myo1        YDFAGRSPNEMTIKKDEIIEIVQKEPSGWWLALKNGAEGWVPATYVTEYKGSTPQTTASS 1174
C.elegans Hum-1     YPYEAQDVDELSFEAGAEIELMNKDASGWWQGKVNNRVGLFPGNYVKE------------ 1100
H.sapiens Myo1e     YAYDAQDTDELSFNANDIIDIIKEDPSGWWTGRLRGKQGLFPNNYVTKI----------- 1108

                                                        Acidic tail region
C.elegans Hum-5     -------------------------------------------    1017
H.sapiens Myo1d     -------------------------------------------    1006
S.pombe Myo1        TNVAAQANNNASPAEVNNLAGSLADALRMRASAVRGSDEEEDW    1217
C.elegans Hum-1     -------------------------------------------    1100
H.sapiens Myo1e     -------------------------------------------    1108
```

Percentage identity matrix of myosins 1d & 1e from *C. elegans*, *H. sapiens* & *S. pombe*

	1.	2.	3.	4.	5.
1: *C.elegans* Hum5	100.00	49.70	37.36	37.46	39.55
2: *H.sapiens* Myo1d	49.70	100.00	36.95	37.16	39.04
3: *S.pombe* Myo1	37.36	36.95	100.00	40.56	41.81
4: *C.elegans* Hum1	37.46	37.16	40.56	100.00	57.84
5: *H.sapiens* Myo1e	39.55	39.04	41.81	57.84	100.00

Percentage identity matrix of *C. elegans*, *H. sapiens* & *S. pombe* myosin 1e TH1 domains

	1.	2.	3.
1: *S.pombe* Myo1	100.00	28.81	31.21
2: *C.elegans* Hum1	28.81	100.00	51.30
3: *H.sapiens* Myo1e	31.21	51.30	100.00

Supplementary Figure 2: Densitometric analysis of anti-mNeongreen western blots of PIP strips incubated with either wild type (white) or S782D phosphomimetic (grey) mNeongreen-HUM1-TH1 domain fusion proteins (shown in Figure 4F).

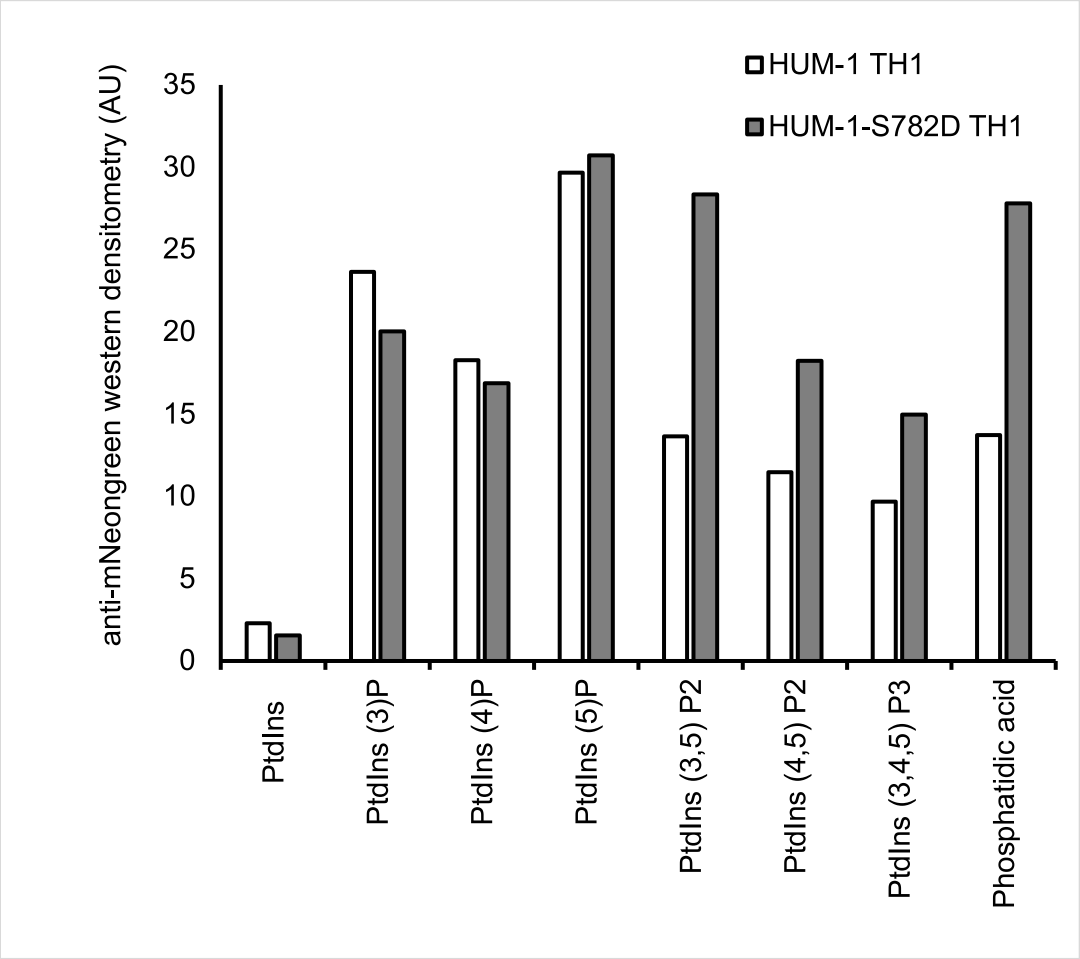

Supplement: Supplementary file 1 — Data S1. Supporting Information. [file CM-82-837-s001.pdf]
